# Multilevel, risk group-oriented strategies to decrease sickness absence in the public sector: evaluation of interventions in two regions in Sweden

**DOI:** 10.1007/s00420-022-01864-6

**Published:** 2022-04-22

**Authors:** Christian Ståhl, Isa Norvell Gustavsson, Ingibjörg H. Jonsdottir, Magnus Akerstrom

**Affiliations:** 1 Department of Behavioral Sciences and Learning, Linköping University, 581 83 Linköping, Sweden; 2 HELIX Competence Center, Linköping University, 581 83 Linköping, Sweden; 3 Department of Health, Medicine and Caring Sciences, Linköping University, 581 83 Linköping, Sweden; 4 The Institute of Stress Medicine, Region Västra Götaland, 413 19 Gothenburg, Sweden; 5 School of Public Health and Community Medicine, Institute of Medicine, The Sahlgrenska Academy at the University of Gothenburg, 405 30 Gothenburg, Sweden

**Keywords:** Sickness absence, Workplace interventions, Process evaluation, Multilevel strategies, Public sector

## Abstract

**Purpose:**

Sickness absence has been identified as needing to be addressed through multilevel interventions, but knowledge regarding optimal design and implementation of such interventions is scarce. The aim of this study was to evaluate the implementation and effects of a large-scale multilevel intervention in the public sector in Sweden.

**Methods:**

The overall effect of the intervention was assessed using mixed-effect models. Sickness absence data (before, and 6 or 12 months after the intervention) for 90 intervention groups and 378 reference groups was retrieved from administrative personnel systems from the two participating regional councils. The implementation processes were evaluated using qualitative content analysis of qualitative interviews conducted at two timepoints.

**Results:**

The results show that the vast majority of implemented measures were on an individual level and the integration of the intervention differed between the two regions. The reception and perception of the intervention activities seem to have been influenced by the implementation process, and how well the interventions were communicated and integrated, both regarding the integration of the different interventions and their integration into the discrete context and existing routines. No short-term overall effects on sickness absence were found.

**Conclusions:**

The results point to the many challenges in implementing complex interventions, especially where organizational measures are involved—including adequate participation by, and communication between, the involved actors, as well as sufficient resources. The results indicate potential learning effects regarding the awareness of organizational factors in sick leave, after implementing and integrating multilevel strategies.

## Introduction

Sickness absence, especially in the public sector, is a major challenge for employers, both in Sweden and in other countries. Even though the relationship between specific working conditions and ill health has been studied for various diagnoses, there is still limited knowledge about how to improve these adverse working conditions and thus decrease the sickness absence among employees (Speklé et al. 2010; Nielsen and Randall 2013; Oude Hengel et al. 2013; Hansen et al. 2019; Burgess et al. 2020; Karanika-Murray et al. 2016). Previous research has recommended multilevel strategies including individual, group, and/or organizational-level interventions to meet the complex challenge of achieving long-term improvements in working conditions (Hasson 2005; Martin et al. 2016). In addition, the importance of incorporating measures on an organizational level in a workplace intervention has been stressed, because at this level, measures can address “the cause of the causes” of work-related illness in the workplace rather than improving the health of individuals (Nielsen and Randall 2013; Cox et al. 2007; Giga et al. 2003; Nielsen et al. 2010a; Kompier and Kristensen 2001; Berg et al. 2017). Despite these recommendations, and despite having identified that the challenge is organizational, individual measures are often still chosen (Martin et al. 2016; Richardson and Rothstein 2008; Sauter and Murphy 2004). Moreover, evaluations of interventions on an organizational level have shown inconsistent results (Gray et al. 2019; Montano et al. 2014; Palmer et al. 2012; Ruotsalainen et al. 2014; Semmer 2006) and the need for more high quality evaluation studies, covering both the process and the outcomes, has been raised (Ruotsalainen et al. 2008).

Since resources for occupational health initiatives are often scarce and the needs are extensive, one increasingly common approach is to develop strategies or interventions directed at groups of individuals who need the support the most, i.e., risk groups. One measure shown to have effect in reducing sickness absence is early contact with occupational health care (Kant et al. 2008). This approach is an example of secondary prevention strategies that aim to prevent initial minor problems from growing into severe problems. Such interventions should preferably be combined with primary prevention strategies, such as work environment improvements, and tertiary prevention, such as return to work (RTW) programs, to maximize the intervention effect. Previous research on determinants of sickness absence has identified different factors that may influence both sickness absence and RTW, which could be used to identify risk groups (Speklé et al. 2010; Hansen et al. 2019; Janssens et al. 2014; Holmgren et al. 2019). However, most previous studies identifying predictors for sickness absence and earlier RTW investigated individual-level predictors, such as symptom severity, previous illness, gender, education, health condition, age, and RTW expectations. Increasing the knowledge on predictors on a workplace or organizational level could help us design more efficient interventions and secondary prevention strategies, in terms of both preventing sickness absence (Hasson 2005; Martin et al. 2016) and reducing the RTW time (Berg et al. 2017; Ahola et al. 2017; Wallensten et al. 2019).

Research focusing on workplaces and organizations has developed guidelines for addressing the issue (Kwak et al. 2019); some studies have also scrutinized existing workplace policies and guidelines and concluded that many of these (which are focused on mental health problems) include primary, secondary, and tertiary preventive strategies, but are often based on outdated evidence (Nexø et al. 2018). Hence, research on the effects and the implementation process of multilevel strategies to prevent sickness absence is needed to provide workplaces and organizations not just with evidence-informed strategies, but also with thorough information on how these strategies could be successfully integrated into daily operations.

Since secondary prevention interventions that simultaneously target individual, group, and organizational levels are still relatively uncommon, it is relevant to report results of their implementation. The aim of this article is to report on one such intervention, which were administered in two Swedish regions. Specific research questions for this study were:

1. How were the different parts of the intervention implemented and integrated into daily practice in the two regions?
2. Did the intervention result in overall changes in sickness absence?

### A risk group-oriented approach

The context of the study is two Swedish regions, which are equivalent to counties, with regional councils responsible for providing public health care and other services. The workplaces included in this study are health care organizations in these two regions.

In 2017, the Swedish Association of Local Authorities and Regions (SALAR), an umbrella organization for Swedish regions, initiated a project to develop a risk group-oriented approach for decreasing sickness absence among public health care and service employees. The project began with screening in five regions and eight municipalities with the aim to identify needs and specific groups at risk for long-term sickness absence (> 60 days). On an individual level, risk groups identified in this process were employees with repeated short-term sickness absence (≤ 14 days), and employees returning to work after longer sickness absences. On a workplace level, the screening identified workplaces with a high level of short-term sickness absence, and workplaces with high or increasing sickness absence, as being at risk.

Based on these screenings, a multilevel intervention approach was developed by SALAR and a consulting company, targeting the employee, group/workplace, and management level. The implemented measures within this intervention were designed by local rehabilitation coordinators (RCs) and intervention leaders (see below) in cooperation with the participating individuals and workplaces to ensure a good fit between the interventional measures and the local context (Karanika-Murray and Biron 2015; McFillen et al. 2013). Two of the authors (IHJ and CS) were involved in the process in an advisory capacity.

The first part of the intervention was targeted towards individuals and was based on a RC function at the workplace. This included regular follow-ups of sickness absence patterns using the employer’s administrative systems to identify employees at risk, in combination with dialogue with managers, human resources (HR), and health care providers. The first part of the intervention then focused on providing support to employees at risk for sickness absence, employees who were currently part-time absent, and employees recently returning to work from a longer sickness absence (> 15 days). The method used primarily consisted of motivational support for the employee, coordinating the sickness absence and RTW process, and reporting of work environment issues at the workplace level if considered relevant. The RCs had a background in HR or health care and all RCs received 2 days of training.

The second part of the intervention was targeted towards the workplace and included an initial screening to identify workplaces with high or increasing rates of sickness absence. The workplaces were contacted by an intervention leader to initiate a dialogue and to map risk factors, to develop specific measures to be carried out and followed up. The interventions were led by one person in each region who received a 2-day training.

The third part of the intervention consisted of a series of workshops aiming to improve cooperation between health care organizations and managers in health care. The purpose was to increase an exchange of knowledge and experiences and develop new ideas and solutions based on problems identified by the participants. The third part of the intervention included six meetings with participation from employers, health care, the Social Insurance Agency, occupational health care, and union representatives. A process leader in each region was responsible for the series regionally.

The intervention was piloted in two regions in the south and north of Sweden (hereafter named Regions 1 and 2) in 2018 and 2019. In Region 1, about half of the healthcare workers in the intervention groups worked within the primary care, 25% within the dental healthcare and the remaining 25% within hospital care. In Region 2, about 75% worked within hospital care and about 25% within the primary care. The pilot implementation was evaluated both by the consulting company which participated in developing the interventions, and by the team of researchers who have authored this article.

## Methods

The study applied a mixed-methods approach to capture the effects and processes of the interventions. The effects were measured based on statistics from administrative personnel systems, and the processes were evaluated using qualitative interviews at two timepoints.

### Process evaluation

The process evaluation was conducted using qualitative interviews at two timepoints. The first wave of interviews was carried out at the beginning of the interventions (autumn 2018), and the second was conducted 1 year later. Participants included managers and staff engaged in the implementation of the interventions. The first wave included 20 interviews (twelve from Region 1, and eight from Region 2), while the second wave included 14 interviews (six from Region 1, and eight from Region 2), i.e., a total of 34 interviews (see Table 1). Documentation about the interventions was collected from SALAR and from the regions.

**Table 1:** Overview of interviews

	First wave	Second wave
Region 1		
Unit manager	X	
Unit manager	X	X
Unit manager	X	
Unit manager	X	X
Unit manager	X	
Unit manager		X
Operations manager	X	X
Intervention owner	X	
HR specialist (intervention owner at second interview)	X	X
HR specialist	X	
HR manager	X	
RC	X	
RC	X	X
Region 2		
Unit manager	X	X
Unit manager	X	X
Unit manager	X	X
Unit manager		X
HR director	X	
Occupational health care representative	X	X
Process leader	X	X
RC	X	X
RC	X	X
Total number of interviews	20	14

*HR* human resources, *RC* rehabilitation coordinator

The interviews in the first wave included questions about local work environment practices and how sickness absence was being managed; policies on sickness absence and rehabilitation; knowledge about and experiences regarding risk factors, preventive work, and rehabilitation; sources of information and support; processes for work adaptations; and questions about the interventions (which parts of the interventions were being applied; how they were applied and integrated with current practices and with the local context; perceived usefulness; and plans for implementation). The second wave of interviews was a follow-up on the first wave and included questions about the extent to which interventions were used as well as their perceived usefulness; and whether they had led to any changes in practices related to sickness absence and work environment management.

Interviews were carried out face to face, audio-recorded, and transcribed verbatim. Qualitative content analyses (Hsieh and Shannon 2005) were conducted using an iterative approach. This started with inductive exploration of the material, which was in the next stage mapped to Nielsen and Randall’s model for process evaluations (Nielsen and Randall 2013), indicating a more directed analysis approach. The inductive exploration corresponded well with this model, which made it adequate as a tool for categorizing the data. The categories are, therefore, based on the model’s focus on context, intervention, and mental models.

### Evaluation of intervention effects on sickness absence

To assess intervention effects on sickness absence for the first and second part of the intervention (i.e., the individual- and workplace-level measures), monthly data from the two regions’ administrative personnel systems were obtained for each intervention group and for the remaining workplaces at the respective departments (i.e., reference groups). In Region 1, data was obtained for the period January 2015 to August 2019 and sickness absence was expressed as the number of absence days per employee on a workplace level (vacation, parental leave, and caring for sick children deducted). In Region 2, data obtained covered the period January 2012 to March 2019. Sickness absence was calculated as the percentage absence on a workplace level based on the number of hours of absence due to sickness, divided by the total number of hours the group was expected to work each month (vacation, parental leave, and caring for sick children deducted). The sickness absence was stratified by short-term (1–14 days) and long-term sickness absence (> 60 days). The intervention was planned to start in September and October 2018 for Region 1 and Region 2, respectively.

In total, 503 workplaces (i.e., dental health care offices, medical wards, surgeries etc.) from ten departments (i.e., higher level organizational units corresponding to geographical areas within the dental health care, medical clinics etc.) were originally included in the study (151 workplaces from six departments in Region 1 and 352 workplaces from four departments in Region 2). In total, 125 of these workplaces received the intervention, while the remaining 378 workplaces served as reference groups. However, eight intervention groups underwent organizational changes right before or immediately after the intervention start and could not be included in the effect evaluation. In addition, 27 intervention groups with very few employees (fewer than ten employees) were also excluded from the evaluation, resulting in 90 intervention groups remaining in the effect evaluation. Descriptive information on these groups and the intervention they received is presented in Table 2. The excluded workplaces did not differ in any significant way from the remaining intervention groups.

**Table 2:** Number of intervention groups, type of intervention, and mean number of participating employees

	Region 1	Region 2	Total
Intervention groups, *n*	52	38	90
Organizational workplace measures, *n*	0	1	1
Individual rehabilitation coordination, *n*	48	32	80
Both organizational and individual measures, *n*	4	5	9
Number of employees receiving individual support per workplace, mean (range)	5.6 (1–19)	3.6 (1–13)	4.7 (1–19)
Percentage of employees receiving individual support per workplace, mean (range)	16 (2.2–42)	13 (1.4–35)	14 (1.4–42)
Employees per intervention group^a^, mean (range)	36 (11–113)	30 (11–118)	33 (11–118)
Employees per reference group^a^, mean (range)	28 (10–79)	24 (10–118)	26 (10–118)

^a^ Calculated as mean of the mean for the monthly number of employees per workplace.

#### Statistical analysis

The measures of sickness absence (i.e., total, short-term (≤ 14 days), and long-term (> 60 days) sickness absence) were tested for normality using the Shapiro–Wilks test and visual inspection of the generated histograms. The normality assumption was deemed to be plausible. In the subsequent analyses, parametric methods were used on untransformed data. The intervention effect was evaluated separately for the two regions due to differences in calculating sickness absence (based on days and hours, respectively), using a mixed-effect model (Proc Mixed in SAS version 9.4; SAS Institute, Cary, NC, USA), with workplace, department, and time (nested within workplaces and departments) as random effects (Akerstrom et al. 2021a). A first-order autoregressive correlation structure (AR (Speklé et al. 2010)) was used to account for correlations between repeated measurements in the same group. In addition, fixed effects for year (continuous) and month (categorical 1–12) were added to the model to control for time trends and seasonality, and an interaction term between a dummy variable for the intervention group (1 = intervention group, 0 = reference group) and a dummy variable for the intervention (0 up to the beginning of the intervention, thereafter 1) was added to analyze the effect. Hypothesis testing for fixed effects was performed using Wald tests, and tests of random effects were performed using likelihood ratio tests.

### Ethical considerations

The interventions described in this article were developed by SALAR, a national public organization, in collaboration with a consulting company. Two of the authors (IHJ and CS) acted as advisors in the process but were not involved in either developing or implementing the interventions. None of the authors have any conflicts of interest, and none of the involved organizations has had any influence over the design, analysis, or conclusion of the study.

Participants in the interview study were invited via email. Participants were given information about the purpose of the study and were assured that participation was voluntary and that they could withdraw their participation at any time. Register data was collected from the regions by the consulting company; personal data, such as names and social security numbers, were replaced with a unique identification number before being distributed to the authors of the present study, to ensure the anonymity of the employees of the participating workplaces.

The project was reviewed and approved by the Regional Ethics Review Boards in Gothenburg (for the effect study, dnr. 2017/887-17) and Linköping (for the process evaluation, dnr. 2018/264-31).

## Results

In total, 52 employees received support from RCs in Region 1, and 37 employees in Region 2. Of those who were offered support, 74% accepted it. The most common diagnostic groups were musculoskeletal and mental disorders. The majority of those who received support had had several prior short-term sickness absence spells but were not commonly absent at the time of the intervention. Workplace support was carried out at four workplaces in Region 1 and at five workplaces in Region 2, reaching 255 and 264 employees, respectively. Six workshops were carried out in each region.

### Process evaluation results

The results from the process evaluation are reported following Nielsen & Randall’s model (Nielsen and Randall 2013), which includes three broad categories: context, intervention, and mental models. These and their subcategories will be described below.

#### Context

The larger context (omnibus context) of the study is described in the Introduction. The discrete context in which the implementation was planned to take place consists of the workplaces and their existing practices regarding how they manage the work environment. Both regions described their work on managing the work environment as being based on a yearly routine, including safety rounds, regular workplace meetings, and yearly manager–employee conversations. Some managers highlighted the continuous character of this work, which occurred daily through dialogue with employees, and emphasized that they, as managers, ought to be available for their employees and act on everyday obstacles.

At the time of reporting (and of the interviews), the guidelines for how to manage sickness absence were similar in the two regions, where sickness absence was reported to first-line managers or the managerial administrative support. The guidelines also stated that managers were to contact employees on sickness absence within 3 days. Employees with repeated short-term sickness absence (more than three occasions in 6 months) were invited to a meeting with their first-line manager. The guidelines further contain policies for prevention, rehabilitation, and RTW; some managers mentioned these policies, while others did not. Managers could also use HR departments and external occupational health services to support them in working with these processes. As for work adaptations, a common statement from managers was that these were difficult to arrange due to “slim” organizations, especially regarding changing and adapting work tasks. Usually, only temporary adaptations, mostly in the form of reduced work hours, were possible. Hence, regular practice in relation to sickness absence was an individual-oriented process with limited leeway to adjust the work context. The role of RCs, as detailed in the description of the intervention, includes the possibility of raising workplace issues when they were identified, which increases the possibility of identifying organizational risk factors. However, it is not certain that identified measures are acted upon, since this may be influenced by the context, including the capacity and resources of the organization.

The context can both facilitate and hinder implementation of interventions. The existing routines can be considered a facilitating aspect, since they provide a structure within which interventions can be introduced to supplement current measures. They could, however, also be hindering if such structures are incompatible with the interventions, e.g., if responsibilities outlined in the interventions do not correspond to the responsibilities in the organization. One example concerns how RCs were meant to interact with managers and what mandate RCs should have, since the RC role was not included in the ordinary structures of the organization. Another contextual issue was problems with recruiting people with relevant competencies to work as RCs; more generally, logistic problems as well as lack of time and resources when introducing new routines were considered hindering factors for implementing the interventions.

#### Interventions

The intervention part of Nielsen and Randall’s model (Nielsen and Randall 2013) includes several subcategories which relate to the *initiation* of the interventions, the *activities*, and the *implementation strategy*.

The project was *initiated* by SALAR (described in the Introduction). As for *activities*, since the project was mainly top-down initiated, several representatives from both regions reported problems in anchoring the project in their organizations, with uncertainties both regarding its purpose and the various roles in it. These uncertainties were also experienced by the managers responsible for carrying out the interventions, which prevented them from communicating the interventions to the employees in an adequate way. Consequently, some employees expressed resistance towards participating, including receiving support from the RCs. Region 1 had a large turnover among managers, which further complicated communication. Both regions largely followed the handbook without significant adaptations to the local context. Among the adaptations made, Region 1 customized communication with the RCs based on managers’ wishes. In Region 2, some adaptations were made to the second part of the intervention (local workplace support), where the handbook suggested that one group should perform the work environment screening, and another group should lead the actual work with implementing changes; in Region 2, it was decided that both the screening and the changes were to be carried out by the same group.

In Region 1, the RCs and the workplace support were separately organized, and therefore, these parties did not interact. In Region 2, there was a collaboration between the RCs and the local workplace support. As RCs generally worked faster with implementation, RCs entered the workplace first, after which local workplace support was added. There were also differences in the two regions regarding the required competencies of RCs, where Region 2 chose people with an HR background, while Region 1 employed health care professionals. In Region 2, RCs and occupational health care staff experienced some problems in finding employees who wanted to participate in the intervention, and the RCs felt insecure in their role. Recruitment was identified as an important issue and something that should have been given more attention before implementation began.

The *implementation of the first part of the intervention* (RCs) was based on different perspectives on the purpose of the RC role and whom the RCs were serving. This intervention was seen as a support primarily for individuals on sickness absence, and secondarily for managers. The task of the RCs was seen as identifying people who are at risk for sickness absence and initiating early interventions, shortening existing sickness absence spells, and helping employees in the RTW process. However, representatives from both regions were slightly unclear about the role, especially regarding the RC’s responsibilities. The selection of individuals to be offered the intervention was made in dialogue with the manager based on sickness absence statistics and the manager’s knowledge of the employees. After initiating contact, the RC carried out an exploratory conversation and formed an individual plan with the employee. In cases where the employee was considered to have the situation under control, no specific measures were taken.

The handbook was generally followed although the templates for conversations were perceived to be cumbersome. The RCs instead chose to develop their own conversations and plans, based on the handbook, which can be considered a local adaptation of the intervention. The types of activities carried out included supportive conversations, help in navigating through measures from the health care or occupational health care systems, contacts with the Social Insurance Agency, and work adjustments.

Overall, the RCs were well received by most managers and employees in both regions. Managers perceived them as helping with the workload and considered it positive that employees could feel seen and listened to. They believed that employees appreciate the contact, especially having an outside professional who may be easier to talk to than their managers. Some managers, however, thought the contact with and feedback from RCs was poor. In Region 1, some discontent was expressed that was generally not reported in Region 2, including managers feeling that their responsibilities had been taken away and they were, therefore, becoming more distanced from their employees. Some managers also did not think that they received sufficient feedback from the RCs due to confidentiality. The first of these points (responsibility) is an example of a situation where existing routines (context) can hinder implementation. The second point (confidentiality) was handled differently in the regions, where Region 2 was very clear that the RCs were representing the employer. This could also explain why fewer employees in this region agreed to participate in the intervention. Managers in both regions reported that some employees felt they were “being questioned” when contacted by the RC, especially those with short-term sickness absence.

In both regions, the RCs had to spend much time explaining the intervention to both managers and employees, since it was not perceived as clear. This could partly explain the skepticism several employees expressed towards the intervention, some even declining it, especially in Region 2. The managers expressed that while the support from the RCs was appreciated by employees, the intervention did not seem to lead to any changes in the workplace. Several managers did, however, think that it could have a long-term effect on sickness absence.

The *implementation of the second part of the intervention* (local workplace support) differed between the two regions: it seems to have been well integrated in Region 2, and largely ignored in Region 1. In Region 1, the position of intervention leader was vacant for a long time, and the intervention was consequently not widely used. Some workplaces started work environment screenings and also suggested some measures, but few of them were conducted. From the interviews, we can conclude that those who carried out measures resisted attributing the work done to the intervention, because they did not receive any special support for this work.

In Region 2, the first intervention leader quit, and a new person was recruited at the end of 2018. This person had a background as an occupational health nurse and as an RC in primary health care, which was described as a strength. Consequently, the work was perceived to be more effective and to progress. At this point, several changes were introduced regarding the work procedures, where the intervention leader carried out the initial work environment screenings on her own, and thereafter added the necessary competencies from occupational health care. Workplaces that received this support described having received help with structuring their work and prioritizing what to focus on. This support was also oriented towards how to continue after the intervention ended. Overall, those involved considered the intervention as very positive.

Some of the measures taken in Region 2 were to revise routines and structures in work environment management, and develop these together with occupational health care. Measures also included risk assessments, workflow revisions, specific measures targeting certain professions, medical controls, ergonomic surveys, and schedule changes. Training focusing on psychosocial health, and on discrimination, threats, and violence in the workplace also took place. Hence, the measures varied quite significantly depending on the identified needs in the specific workplace. Finding the root cause of problems was described as the crucial aspect of the intervention and it was vital to have consensus on both problems and measures to address them. The representatives described changes in the work environment management in the workplaces and reported that awareness of the issues had increased. In Region 2, the implementation strategy worked well, with adequate participation from employees and managers. Moreover, communication about measures, and their implementation, seems to have been well adapted to local conditions.

The *implementation of the third part of the intervention* (workshops) was affected by the fairly vague purpose of the intervention. This intervention served as an arena for discussion, but the outcomes were highly dependent on whether the participants used it and also on organizational prerequisites for moving from discussion to concrete action.

In Region 1, the experiences of the workshops were mixed. Workshops were considered as a meeting place for the central actors in rehabilitation processes and as creating extended networks. However, criticism was raised about the limited time available for the intervention so that discussions remained superficial and, hence, did not lead to any concrete measures. In Region 2, participants in the workshop series were relatively positive and described the contents of the workshops as concrete and the workshops as useful for identifying areas that needed improvement. The workshops resulted in guidelines for creating health-promoting workplaces, and working with sickness absence and rehabilitation processes. The participants described the workshops as an important piece of the larger project whose interventions all interact.

As for the *integration of the interventions*, Region 1 treated the RCs and the local workplace support (to the extent that this was carried out) as parallel interventions and there was no collaboration between them. The RCs in the region described that they identified problems in the workplaces, but that they did not have anyone to report to. Such issues were raised on several occasions to the management, but nothing changed. Representatives also raised the lack of integration between the different interventions as a problem.

In Region 2, the interventions seem to have worked in concert, as the implementation strategy focused on integration. The quicker intervention (the first part of the intervention, involving the RCs) entered the workplaces first, followed by the slower local workplace support intervention (the second part of the intervention). The RCs had dialogue with the local workplace support, reporting what they had learned. They participated in meetings to add their perspectives, which added information that could be used in work environment management. Representatives from Region 2 perceived the integration of the interventions as successful as it made it possible to work on individual and organizational levels simultaneously. The workshop series was considered an arena for integrating the different parts. The representatives in Region 2 also described a well-integrated team working closely together, which led to a constant flow of information and work procedures. Occupational health care was also involved to a large extent.

#### Mental models

“Mental models” refer to how people think about certain phenomena, where interventions that can be proven to have an effect on such models can be said to have a learning component. In Region 1, no specific changes in awareness of the different issues influencing sickness absence were reported. This is possibly related to the lack of focus on workplace aspects, as this region primarily implemented the first part of the intervention (RCs) which focused on individual support. The integration between individual support and the more organizationally focused interventions was poor, which reasonably limits the impact on organizational learning, and on recognizing non-individual causes for sickness absence.

In Region 2, the representatives reported that working with the interventions led to increased awareness about work environment issues, both among employees and among managers, indicating a change in mental models. They also reported having seen concrete changes in the workplaces regarding work environment management and increased focus on such issues, which also indicates how these mental models increased the readiness for change, as the management and employees had already started translating their new awareness into action. Furthermore, Region 2 chose to continue using the interventions, albeit with slight modification, after the end of the project, which is an indication of changed mental models, where the staff could see the value of continuing with changing the workplace.

Hence, the effect on mental models seems to have been influenced by how well the interventions were communicated and integrated, regarding both the integration of the different interventions and how well they were integrated into the discrete context and existing routines.

### Intervention effects on sickness absence

**Fig. 1:**
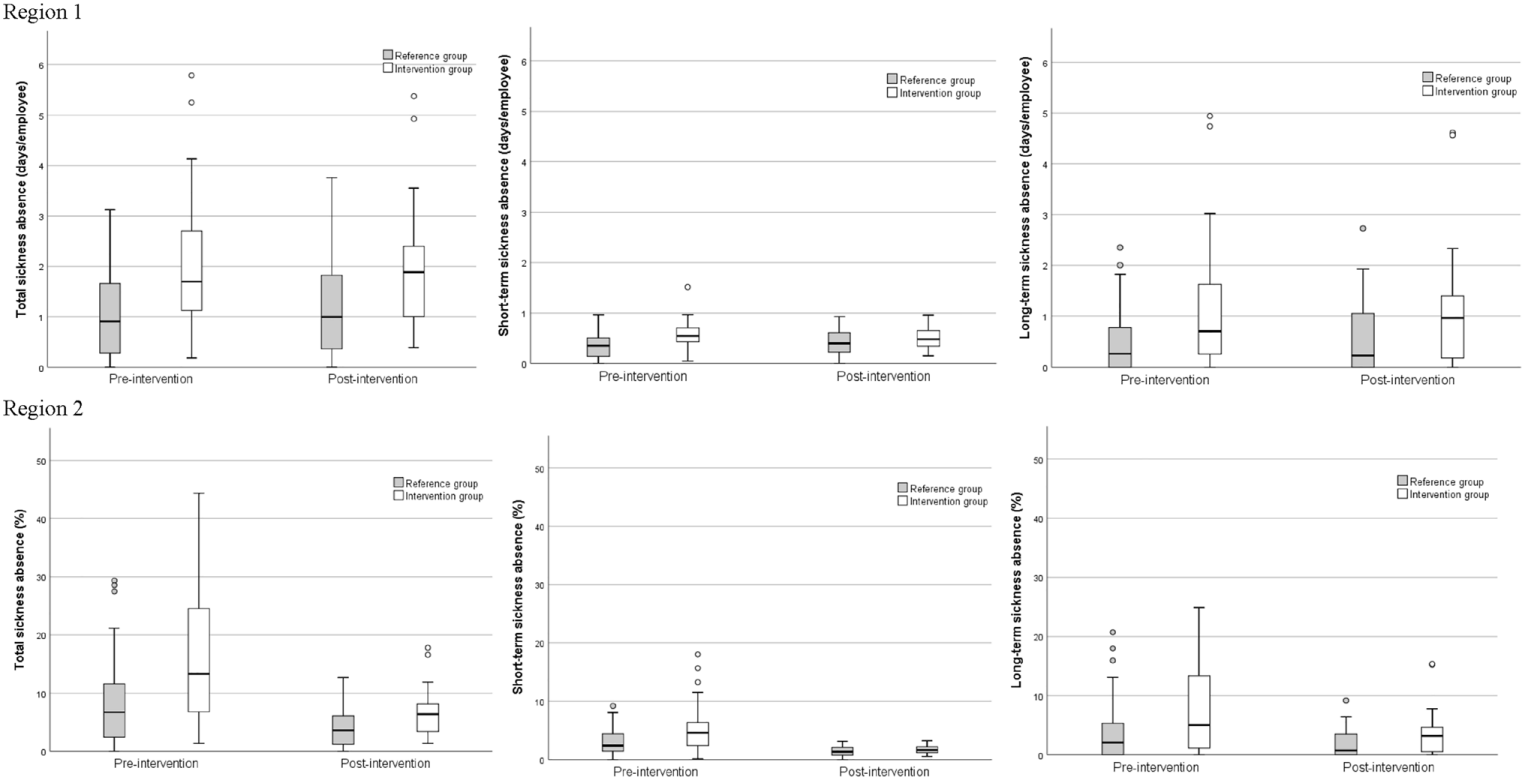
Distribution of average individual workplace levels of total, short-term (≤ 14 days), and long-term (> 60 days) sickness absence pre- and post-intervention for intervention reference groups

The distribution of individual workplace average levels of sickness absence, pre- and post-intervention, among intervention groups participating in the first two interventions and reference groups is presented in Fig. 1.

For Region 1, the intervention groups (*n* = 52) participating in the first and/or second intervention (i.e., the interventions on an individual and/or a workplace level) had somewhat higher total and short-term sickness absence compared to the reference groups (*n* = 99) (*β* = 0.82, 95% confidence interval (CI) 0.21–1.4, *p* = 0.007, and *β* = 0.20, 95% CI 0.13–0.27, *p* < 0.001, respectively), but no difference was seen between the intervention groups and reference groups in Region 2 (Table 3). In addition, a trend for an overall decrease in total and long-term sickness absence was seen for all workplaces in Region 1 during the time of the intervention (*β* = − 0.14, 95% CI − 0.29 to 0.001, *p* = 0.05, and *β* = − 0.05, 95% CI − 0.14 to 0.04, *p* = 0.07, respectively) (Table 3).

**Table 3:** Effects of time trends (year), seasonality (month), group (intervention or reference), and intervention status (pre- and post-intervention) on sickness absence, by region

	Region 1						Region 2					
	Number of observations (*n*)	Number of departments (*N*)	Number of workplaces/intervention group (*N*)	Fixed effects^a^	Estimate (95% CI)	*p* value	Number of observations (*n*)	Number of departments (*N*)	Number of workplaces/intervention group (*N*)	Fixed effects^a^	Estimate (95% CI)	*p* value
Sickness absence^b^												
Total	5555	6	151/52	Intercept	1.2 (0.73 to 1.7)	< 0.001	12,688	4	314/38	Intercept	15.6 (− 1.1 to 32.3)	0.07
				Year	0.0 (− 0.05 to 0.05)	0.9				Year	− 1.3 (− 4.5 to 1.8)	0.4
				Month	–^c^	< 0.001				Month	–^c^	< 0.001
				Group	0.82 (0.21 to 1.4)	0.007				Group	− 0.23 (− 5.4 to 4.9)	0.7
				Intervention	− 0.14 (− 0.29 to 0.001)	0.05				Intervention	0.09 (− 1.8 to 2.0)	0.4
				Intervention × group	0.07 (− 0.11 to 0.26)	0.4				Intervention × group	− 1.5 (− 4.5 to 1.6)	0.4
Short-term (≤ 14 days)	5555	6	151/52	Intercept	0.41 (0.31 to 0.50)	< 0.001	12,688	4	314/38	Intercept	9.7 (− 2.3 to 21.7)	0.1
				Year	0.01 (− 0.004 to 0.02)	0.2				Year	− 1.1 (− 3.2 to 1.0)	0.3
				Month	–^c^	< 0.001				Month	–^c^	< 0.001
				Group	0.20 (0.13 to 0.27)	< 0.001				Group	0.64 (− 0.41 to 1.7)	0.5
				Intervention	− 0.02 (− 0.08 to 0.03)	0.1				Intervention	− 0.02 (− 1.4 to 1.3)	0.7
				Intervention × group	− 0.02 (− 0.08 to 0.05)	0.6				Intervention × group	− 0.35 (− 2.4 to 1.7)	0.7
Long-term (> 60 days)	5555	6	151/52	Intercept	0.50 (− 0.03 to 1.0)	0.06	12,688	4	314/38	Intercept	3.1 (0.64 to 5.6)	0.01
				Year	0.01 (− 0.05 to 0.07)	0.7				Year	0.35 (− 0.13 to 0.83)	0.2
				Month	–^c^	< 0.001				Month	–^c^	< 0.001
				Group	0.47 (− 0.18 to 1.3)	0.2				Group	− 0.77 (− 5.0 to 3.5)	0.6
				Intervention	− 0.05 (− 0.14 to 0.04)	0.07				Intervention	− 0.05 (− 1.1 to 0.96)	0.3
				Intervention × group	− 0.02 (− 0.14 to 0.10)	0.7				Intervention × group	− 0.73 (− 2.4 to 0.91)	0.4

*CI* confidence interval.

^a^Year (continues, 1–8), month (class variable, 1–12), group (intervention group = 1, reference group = 0), intervention (class variable, 0 until the intervention start, then 1), intervention*group (interaction term used to investigate the intervention effect)

^b^Based on days of absence/employee for Region 1 and hours of absence (%) in Region 2

^c^Estimates for month 1–12 not shown because of limited space

#### Overall intervention effects on sickness absence

No overall intervention effects on the total, short-term (≤ 14 days), or long-term (> 60 days) sickness absence could be seen for the intervention groups participating in the first and/or second intervention in Region 1 or 2 (Table 3).

Furthermore, stratifying the results for intervention groups receiving only individual rehabilitation coordination and for workplaces receiving individual rehabilitation coordination in combination with organizational workplace measures did not change the overall result.

## Discussion

This study illustrates the complexities of conducting preventive interventions which focus on multiple levels, in this case the individual and organizational levels. It also illustrates how differently the same intervention can be implemented in different contexts, and how this relates to interactions and communications between the various actors involved.

The interventions were developed through an interactive process between a national organization, consultants, and representatives from different regions, but the design and preparation did not involve the employees who would implement them and be most affected by them; likewise, unions’ involvement was limited to the workshop series. When implementation commenced, consequently, the interventions were considered top-down initiatives. The implementation included participants from the different workplaces to varying degrees, where such involvement facilitated a more purposeful adaptation to fit the local context. Insufficient stakeholder involvement and contextual restraints, such as production pressures, has been identified in the literature as potentially hampering interventions (Cole et al. 2009; Franche et al. 2005).

In Region 1, the implementation primarily focused on the individually oriented RC part of the intervention, while Region 2 to a larger extent adapted and integrated the multilevel approach of the three parts of the intervention, and, therefore, also included the workplace level. This latter approach seems to have had a stronger impact on the mental models of people involved and resulted in a more developed perspective on the types of factors that can influence sickness absence, including work environment issues. A key for this development was the integration of the individual and the organizational interventions. Only implementing individual support, as in Region 1, does not seem to have effected changes in routines or work environment management, while combining such support with a workplace component facilitated a broader approach towards working with prevention, which has also been reported by others (Hasson 2005; Martin et al. 2016). The RCs were able to identify organizational issues through the individual cases, which then could be picked up by the organizational workplace support intervention. In Region 1, where this latter structure was missing, there was no-one to receive this communication/information when RCs identified such factors, implying that organizational factors remained unresolved.

The qualitative results from Region 2 indicate that the intervention may have been the start of a development process in the workplaces, where sickness absence was treated as both an individual and an organizational issue, and as an issue that calls for measures on both these levels. The results are interesting, because they illustrate how a multilevel intervention, implemented through participative methods, is more likely to be integrated in the daily operations; this is an example of an adaptive and developmental learning process (Ellström 2006). Therefore, in Region 2, the intervention seems to have contributed to a development-oriented learning environment which may lead to long-term organizational learning and a long-term impact on routines within the regular operations of the organization (Fuller and Unwin 2004).

The effect evaluation did not show any significant effects on sickness absence for either of the regions. Varying results for organizational-level workplace interventions have also been reported by others (Gray et al. 2019; Montano et al. 2014; Palmer et al. 2012; Ruotsalainen et al. 2014; Semmer 2006). To understand these inconsistencies, the use of a mixed-methods approach that includes qualitative process data, as in this study, has been recommended to understand how and why an intervention does or does not work (Nielsen and Randall 2013; Egan et al. 2007; Kristensen 2005; Nielsen et al. 2010b). With this approach, one of the two regions was found to have largely failed in implementing measures on an organizational level. In addition, even though the other region more successfully integrated the intervention on an individual and organizational level, relatively few measures were implemented on an organizational level and this most probably was not sufficient to affect the overall result, since, to gain long-term positive effects, measures on an organizational level are recommended (Nielsen and Randall 2013; Cox et al. 2007; Giga et al. 2003; Nielsen et al. 2010a; Kompier and Kristensen 2001; Berg et al. 2017). Difficulties in implementing measures on an organizational level have been shown from another large-scale intervention in the public sector in Sweden (Severin et al. 2021) resulting in no, or limited, effects on the employees’ working conditions and health (Akerstrom et al. 2021b). Therefore, efforts must be made to change the approach, from merely providing measures on an individual level to also including measures on an organizational level when adapting systematic strategies for decreasing sickness absence among employees. This can be done by securing an active participation and communication between the involved actors, as well as securing sufficient resources within the organization for conducting such preventive measures. Earlier evaluations of organizational-level interventions have shown that process facilitators could be used to support the transformation from individual-level measures to organizational-level measures in work environment interventions (Akerstrom et al. 2021a; Härenstam et al. 2019). However, to fully succeed, the process facilitators need to shift between the support and expert role in a complex work environment intervention depending on the workplace capacity for change (Wikström et al. 2022).

Other possible reasons for the relative lack of an intervention effect could be due to the use of sickness absence as the single effect measure. Other outcome measures, such as employee turnover or different work environment measures, may be more appropriate for capturing intervention effects compared to measuring sick leave only. In addition, the somewhat limited follow-up time in this study should be noted: the interventions began in 2018, and follow-up ended in August 2019 for Region 1 and in March 2019 for Region 2. This may potentially have affected the possibility to evaluate the total effect of the intervention as it is reasonable to assume that especially organizational effects take longer to develop.

### Methodological considerations

This study was based on a mixed-methods approach, where we combined a register study with document studies and with interviews conducted at two timepoints. This approach makes it possible to study both effects and processes, which can be considered a strength. For the quantitative material, the short follow-up time for measuring intervention effects is a weakness as the outcomes can be expected to develop over a longer period than that covered by the available data. In Region 1, the trend for an overall decrease in total and long-term sickness absence for all workplaces could, to some extend been explained by regression to the mean in both the intervention and reference group. In addition, the effect evaluation was limited to evaluating effects on sickness absence on an organizational level in accordance with multilevel strategy. Hence, positive effects may still have been achieved for individual employees receiving the RCs’ support.

For the qualitative material, we chose to compare results from the two regions rather than on a workplace level, since the interventions were planned and implemented on the regional level. While variation can be expected also between workplaces, this was not our unit of analysis. Interventions focusing on identifying risk groups may have a potential stigmatizing effect by singling out people with health problems, which the fact that some workers refused the intervention could indicate. It is possible that including data from those who refused the intervention could have added important insights, although this was not possible in the current study.

The interventions evaluated in this study was initiated and performed by a third party in a collaboration with the workplaces, and the research group had no part in carrying out the intervention nor deciding on which data variables that were available for analysis. Several aspects regarding contextual information related to work conditions and more in-depth information about the individuals receiving the intervention was not available for the research group. Thus, research questions related to how different contextual and individual factors plausibly could affect sick leave could not be answered in this study.

## Conclusions

Combining and integrating preventive strategies on an individual and an organizational level can result in stronger awareness of the multifaceted causes of sickness absence and contribute to organizational development and learning, which may lead to long-term changes in workplaces’ approaches to workplace sickness prevention. Although we did not see any short-term effects on sickness absence in this study, the results related to learning within the organizations regarding the awareness of integrating interventions are promising. The results also point to the many challenges in implementing complex interventions, especially where organizational measures are involved—including adequate participation by, and communication between, the involved actors, as well as sufficient resources.

## Data Availability

The data sets generated during and/or analysed during the current study are available from the corresponding author on reasonable request.
